# ILSI Brazil International Workshop on Functional Foods: a narrative review of the scientific evidence in the area of carbohydrates, microbiome, and health

**DOI:** 10.3402/fnr.v57i0.19214

**Published:** 2013-02-07

**Authors:** Marie E. Latulippe, Agnès Meheust, Livia Augustin, David Benton, Přemysl Berčík, Anne Birkett, Alison L. Eldridge, Joel Faintuch, Christian Hoffmann, Julie Miller Jones, Cyril Kendall, Franco Lajolo, Gabriela Perdigon, Pedro Antonio Prieto, Robert A. Rastall, John L. Sievenpiper, Joanne Slavin, Elizabete Wenzel de Menezes

**Affiliations:** 1 ILSI Europe, Brussels, Belgium; 2 Department of Nutritional Sciences, Faculty of Medicine, University of Toronto, Toronto, Ontario, Canada; 3 Swansea University, Swansea, United Kingdom; 4 McMaster University, Hamilton, Ontario, Canada; 5 The Kellogg Company, Battle Creek, MI, USA; 6 Nestlé Research Center, Lausanne, Switzerland; 7 School of Medicine, Hospital das Clinicas-ICHC, University of São Paulo, São Paulo, Brazil; 8 Department of Microbiology, Perelman School of Medicine, University of Pennsylvania, Philadelphia, PA, USA; 9 Instituto de Ciências Biológicas, Universidade Federal de Goiás, Goiania, Brazil; 10 St. Catherine University, St. Paul, MN, USA; 11 Faculty of Pharmaceutical Sciences, University of São Paulo and Food and Nutrition Research Center (NAPAN), São Paulo, Brazil; 12 CERELA–CONICET, Universidad de Tucumán, Chacabuco, Argentina; 13 Abbott Nutrition, Columbus, OH, USA; 14 University of Reading, Reading, United Kingdom; 15 McMaster University and St. Michael's Hospital, Toronto, Ontario, Canada; 16 Department of Food Science and Nutrition, University of Minnesota, St. Paul, MN, USA

**Keywords:** fiber, gut health, prebiotic, glycemia, satiety, carbohydrates, weight, mood, cognition, fructose, biomarkers, functional food, microbiome

## Abstract

To stimulate discussion around the topic of ‘carbohydrates’ and health, the Brazilian branch of the International Life Sciences Institute held the 11th International Functional Foods Workshop (1–2 December 2011) in which consolidated knowledge and recent scientific advances specific to the relationship between carbohydrates and health were presented. As part of this meeting, several key points related to dietary fiber, glycemic response, fructose, and impacts on satiety, cognition, mood, and gut microbiota were realized: 1) there is a need for global harmonization of a science-based fiber definition; 2) low-glycemic index foods can be used to modulate the postprandial glycemic response and may affect diabetes and cardiovascular outcomes; 3) carbohydrate type may influence satiety and satiation; glycemic load and glycemic index show links to memory, mood, and concentration; 4) validated biomarkers are needed to demonstrate the known prebiotic effect of carbohydrates; 5) negative effects of fructose are not evident when human data are systematically reviewed; 6) new research indicates that diet strongly influences the microbiome; and 7) there is mounting evidence that the intestinal microbiota has the ability to impact the gut–brain axis. Overall, there is much promise for development of functional foods that impact the microbiome and other factors relevant to health, including glycemic response (glycemic index/glycemic load), satiety, mood, cognition, and weight management.

Carbohydrates are one of the three macronutrients, which contribute to energy intake. While broadly composed of carbon, hydrogen, and oxygen, they occur in a wide variety of forms that may be classified by chemical structure or physiological effect. These effects are wide- ranging, from impacts on blood glucose levels to alteration of the microbiome. The 11th ILSI Brazil International Workshop on Functional Foods held in São Paulo, Brazil, on 1–2 December 2011, ‘Carbohydrates, Microbiome and Health,’ focused on several types and effects of dietary carbohydrate. The theme ‘carbohydrates’ often stimulates controversial discussion because of conflicting evidence and varying scientific views on their health impacts. The goal of the event was to present consolidated knowledge and scientific advances in recent research on the relationship between carbohydrates and health. The topics of fiber, fructose, glycemic index, mood, satiety, and gut microbiota were covered. This marked the first workshop in the series since the adoption of a definition for dietary fiber by the Codex Alimentarius Commission in 2009 and, as such, presented an opportunity for issues surrounding the implementation of the Codex definition and health benefits of fiber to be discussed by a body of scientific and regulatory experts who are preeminent in the field. Representatives from academia, industry, and regulatory agencies attended the event, and experts from Brazil, Switzerland, Canada, Argentina, the United States, and the United Kingdom presented the state of the science. The workshop was facilitated by professors Antonio Herbert Lancha Junior and Elizabete Wenzel de Menezes, and it was coordinated by Professor Franco Lajolo, all from the University of São Paulo, São Paulo, Brazil.

## Presentation summaries

### Dietary fiber: background, concepts, and definitions

#### Dr. Anne Birkett, Kellogg Company

Dietary fibers are carbohydrates that are not digested within the upper gastrointestinal tract and pass to the lower gastrointestinal tract (large intestine), where they are available for fermentation. Dietary fiber, and its relationship with health and disease, has been the subject of much research and discussion throughout the 20th century. In addition, fiber-rich foods have been recognized for centuries for their association with intestinal health. For example, in the 4th century BC, Hippocrates championed the benefits of wheat with bran to help keep the large intestine healthy. Since the 1970s, a number of important developments have occurred in the field of fiber, spanning disease/health association, definition, measurement, ingredient design, material characterization, mechanisms for physiological effects, and development of nutritional recommendations.

Dietary fibers represent a large family of components. Collectively, these components are carbohydrates, are neither digested nor absorbed in the upper gastrointestinal tract, and show some physiological effect. As a broad family of components, many differences exist among them, including structure (e.g. degree of polymerization [DP], linear or cyclic architecture) and physical and chemical properties (e.g. viscosity, solubility); thus, they can have very different physiological effects and metabolic consequences. Because of the wide variety of materials/ingredients that can be considered as dietary fibers, there is a clear need for a common understanding of dietary fibers supported by an authoritative global definition.

A number of authoritative statements/definitions are available relating to dietary fiber. In 1997, the Food and Agriculture Organization of the United Nations recommended to classify carbohydrates according to their DP (DP 1–2: sugars; DP 3–9: oligosaccharides; DP >10: polysaccharides), not according to origin (i.e. whether they are intrinsic or extrinsic) or solubility (1). Fiber definitions are available from the Institute of Medicine (2), Health Canada (3), the American Association of Cereal Chemists (4), and the European Commission (5), to name a few; however, these primarily have regional impact. The Codex Alimentarius Commission promotes international coordination of food standards, guidelines, and codes of practice. In 2008, Codex developed a definition of dietary fiber (6), which listed three categories of carbohydrate polymers that are not hydrolyzed by the endogenous enzymes in the small intestine of humans: those naturally occurring in food, those isolated from food, and those synthesized. The last two categories have the obligation to show a physiological effect of benefit to health to be considered a dietary fiber. This Codex definition was officially adopted in 2009 with a minor footnote revision (7); however, its attempt to create a globally harmonized definition has not yet reached that goal because Codex left two important aspects of the definition unclear and open for interpretation. First, the definition specifically recognized polymers of DP 10 and above in the body of the definition, relegating polymers with DP 3–9 to a footnote, with their inclusion to be decided by national authorities. The distinction between polymers around DP 9–10 was likely influenced by historical assumptions related to dietary fiber measurement and physiological impact. On the contrary, arguments for inclusion of DP 3 and above (i.e. no distinction at DP 10) include lack of readily applicable methods to clearly distinguish fiber below or above DP 10, and both polymers with a DP between 3 and 9 or >10 can show physiological effects generally associated with dietary fiber. Second, the physiologic effects of benefit to health, and the criteria for their substantiation, remained undefined in the Codex definition. These aspects were discussed during an ILSI session at the Ninth Vahouny Fiber Symposium (8).

Even though Codex made a step forward in attempting to establish a global definition on dietary fibers, it is clear that much remains to be done at both the global and national levels. Indeed, support should be provided to guide national authorities to correctly implement the Codex dietary fiber definition regarding the aspects on DP 3–9 and physiologic effects. A harmonized global definition is fundamental to help further research, method development, and regulation.

### Physiological effects of dietary fiber

#### Dr. Joanne Slavin, University of Minnesota

Dietary fiber is the carbohydrate that is resistant to digestion and absorption in the upper digestive tract. Although fiber was traditionally considered inert, it is now appreciated that fiber has links to health promotion and disease prevention. Originally, effects were thought to be related to solubility, but recent findings indicate that fermentability, viscosity, and other properties may be important (9).

The Institute of Medicine recommends a dietary fiber intake of 14 g/1,000 kcal (10). Interpretation of this recommendation is challenging. For example, recommendations for children are too high under this rule. Although there is no upper limit for fiber intake, overconsumption of added fibers may produce adverse gastrointestinal symptoms (11); however, intake of naturally fiber-containing foods is likely to be self-limiting.

The recommendation for fiber is based on evidence for reduced cardiovascular disease (CVD) risk and lower body weights in prospective cohort studies (12, 13). The mechanism for CVD risk reduction is thought to be lowering of serum cholesterol, delayed nutrient absorption (increased insulin sensitivity and decreased triglycerides), decreased hypertension, and effects of phytochemicals that travel with fiber. Higher dietary fiber intakes are also linked to lower blood pressure, less diabetes, and improved gut health. The mechanism for an impact on body weight is less clear but may be a complex interaction of hormonal effects (lower postprandial glycemia, decreased insulin secretion), effects of the food itself (mastication, satiation), and colonic effects (e.g. fermentation) (14). Both fiber content and food structure seem to have some impact on satiety. Studies indicate that whole fruits or vegetables result in higher satiety and lower subsequent intake than if provided in a processed form (15, 16). There is some suggestion that fiber impacts gut health, although the term ‘gut health’ is in itself poorly defined. It is well-accepted that fiber improves laxation, and there is some evidence that fiber fermentation by gut microbiota provides physiological benefits, including increased mineral absorption (17), stimulation of beneficial microbes (18), decreased survival of pathogenic bacteria, and nourishment of colonocytes with short-chain fatty acids (SCFAs) (19).

Fiber intakes are generally below recommended levels, with American consumption of fiber about half the recommended 25 g/day for females or 38 g/day for males based on 14 g/1,000 kcal. Fiber intakes for children and the elderly are particularly poor in comparison with the guidelines. For these reasons, the 2010 Dietary Guidelines for Americans list fiber as a ‘nutrient of concern’ (20). In Brazil, the average ingestion of dietary fiber was estimated to be 15.4 g/day in a 2002–2003 survey (21) and 12.5 g/day in a 2008–2009 survey (22). Therefore, the slight increase noted in the beginning of the 2000s did not continue throughout the decade.

Because most food sources are not particularly high in dietary fiber, fibers are often added to popular foods, including cereals, breads, dairy products, and beverages. Added fibers have a number of demonstrated benefits but vary greatly in their physiologic effects (Table 1). Public health advice, including the 2010 Dietary Guidelines for Americans, supports consumption of high-fiber foods, including whole grains, legumes, vegetables, and fruits, to increase needed dietary fibers. Added fibers can also be incorporated into the diet to assist in achieving recommended intake levels, and a variety should be consumed for the optimal range of physiologic benefits.

**Table 1:** Benefits of functional fibers

Added/isolated fiber	Beneficial role	Primary source
Cellulose	Laxation	Plant foods
Guar gum	Blood lipid lowering	Guar bean (legume)
	Attenuates blood glucose response	
Inulin/oligofructose/fructo-oligosaccharides	Prebiotic	Chicory root
	Calcium absorption	Jerusalem artichoke
		Synthesize from simple CHO
β-Glucan and oat bran	Blood lipid lowering	Oats and barley
	Attenuates blood glucose response	
Pectin	Blood lipid lowering	Plant foods
	Attenuates blood glucose response	
Polydextrose	Laxation	Synthesized from dextrose (glucose)
	Prebiotic	
Psyllium	Laxation	Psyllium husk (plant)
	Blood lipid lowering	
Resistant dextrins	Blood lipid lowering	Corn and wheat
Resistant starch	Attenuates blood glucose response	Plant foods
Soluble corn fiber	Laxation	Corn

### The glycemic response and health: findings from the ILSI workshop and beyond

#### Dr. Julie Miller Jones, St. Catherine University

Dietary interventions that lower the glycemic index (GI) and/or the glycemic load (GL) of the diet are known to improve fasting blood glucose in individuals with impaired blood glucose control, such as those with diabetes (23). There is currently a lot of controversy in the public and the scientific press regarding the GI and GL concepts, which are complex but may be potentially useful tools to evaluate the glycemic response of the diet as a strategy for maintaining a balanced diet and lifestyle. To provide a clear overview on findings linking glycemic response with health outcomes, the Dietary Carbohydrates Task Force of ILSI Europe commissioned a meta-analysis of intervention studies in this area (24, 25), as well as a workshop and finally a concise monograph (26), to ensure a correct understanding and application of such concepts.

Dietary carbohydrates represent a wide variety of ingredients that do not provide the same blood glucose response. Some may be quickly digested and released in the bloodstream (e.g. sugars and starches), some may be slowly digested but available for a longer period of time in the blood, and finally others are non-available carbohydrates that can be fermented further down in the colon. The blood glucose response therefore depends on the type of carbohydrates ingested, and this response to a specific ingredient can be measured and compared with glucose or a standard food (such as bread): this is called the GI of a tested ingredient. To correctly measure the GI of a food, the 50 g of available carbohydrates is compared with 50 g of glucose or available carbohydrate in a standard food, meaning that it is not a comparison of the same amount of food (27).

There are some additional confounders inducing a variability of the results, such as baseline blood glucose, person-to-person variation, and a variation across time for the same person. The GI of a food can also depend on the way it is cooked (e.g. warm vs. cool potatoes), the degree of maturity (e.g. green vs. mature banana), or the type of the ingredient (e.g. fine flour vs. coarse flour) (28). Some other food components such as lactate, phytates, and tannins may impact the GI or the nutrition profile of the meal (fiber or fat content), which may slow down the digestion. Not only the food combination (29), but also the meal frequency, speed of eating, amount of chewing, the size of the meal, the rate of gastric emptying, the physical fitness of the consumer, and the previous meals can all be confounding parameters to assess the glycemic response.

The GL is another measure of the glycemic response of a food, which takes into account the amount of available carbohydrate in a portion of food eaten and how frequently it is eaten. The glycemic glucose equivalent (GGE) may be the most usable unit for the consumer. Indeed, the GGE gives the theoretical amount of glucose that would give that glycemic response of a specific amount of a specific ingredient (30).

The association of a low GI or GL diet with a variety of health endpoints has been postulated; it has been particularly shown to improve fasting blood glucose in people with impaired blood glucose control, and to improve insulin sensitivity in non-diabetic as well as overweight, obese, and subjects with type 2 diabetes (31). Lowering the GI of a diet has also been shown to be effective in some studies in decreasing low-density lipoprotein cholesterol and reducing glycated proteins concentrations, such as hemoglobulin A1C. There is also some suggested evidence with satiety, weight management, coronary disease, cancer, or exercise, but the results are inconsistent. Indeed, in studies examining satiety, it is difficult to differentiate the effects of higher fiber intake itself (used to lower the GI of the diet) from the effect of a lower postprandial glycemia *per se*, which provokes an effect on satiety. Concerning any impact on body weight, there is some evidence that a lower GI is associated with a reduction of body weight, as presented in the Diogenes study (32). However, more studies are needed to understand whether it is the modulation of the GI *per se* or specific ingredients (such as proteins, as seen in the Diogenes study) that particularly contribute to that effect.

The degree of the association between the glycemic response and these health indicators varies among studies and is influenced by different factors such as body mass index, gender, or baseline blood glucose levels. It could be interesting to see whether there is any possible association with the microbiome.

Overall, it seems to be quite difficult to inform consumers of this concept because it may not work in mixed meals and is dependent of confounding factors such as previous meal effects, and the same foods can have different GIs depending on cooking time. However, despite the controversies, it may still be a good dietary tool for individuals with diabetes to guide them in their food choice.

### The glycemic index and dietary uses/applications in diabetics and non-diabetics

#### Dr. Livia Augustin and Prof. Cyril Kendall, University of Toronto

It was traditionally believed that postprandial blood glucose responses were determined by whether simple or complex carbohydrates were consumed. Over time, increasing experimental evidence has questioned this classification and given rise to the concept of the GI. The GI concept suggests, as an extension of the dietary fiber hypothesis first proposed by Burkitt and Trowell (33), that certain carbohydrates, by virtue of their rate of digestibility and absorption, may provide a strategy to prevent and manage chronic diseases such as diabetes and coronary heart disease (CHD). The GI is a classification of carbohydrate foods based on their blood glucose rising potential (23). Thus, the GI differentiates carbohydrate-rich foods that result in a lower rise (low GI foods) from those that result in a larger rise of postprandial blood glucose (high GI foods). The GI for each food is calculated as the relative glycemic response of the test food compared to the glycemic response of a standard food (e.g. white bread or glucose) given in isoglucidic amounts to the same subject on two different occasions. Therefore, each subject acts as his or her own control. This means that any subjective characteristics (e.g. physical fitness, gastrointestinal motility) would affect both the test and control glycemic responses, which therefore would not significantly affect the final ratio which is the GI value. GI testing follows a strict methodology that also includes controlling for meal quality the day prior to the study and physical activity before and during the study. Low GI diets have been shown to reduce the risk of type 2 diabetes (34, 35), CHD (36, 37), CHD risk factors (38, 39), and some types of cancers (40–43).

Indeed, in clinical trials, low GI foods have been shown to have beneficial effects on blood glucose control (31, 34) and hyperinsulinemia as well as blood lipids (44) and satiety (45, 46), which are of relevance in a diabetogenic and obesogenic environment (47). Low GI diets have not yet been tested in diabetes prevention; however, when converting high GI meals to low GI meals by use of an α-glucosidase inhibitor (acarbose), in high-risk individuals, a significant 25% reduction in the progression to diabetes and a 49% relative reduction in CVD events (equivalent to 2.5 absolute risk reduction) over 3.3 years were found in the test group (48). Low GI diets may result in benefits also in healthy individuals. A number of studies suggest direct associations of both fasting and postprandial hyperglycemia with all-cause and CVD mortality in non-diabetic populations (36, 37, 49, 50). Furthermore, in non-diabetic subjects with normal glucose tolerance, beta cell function was 60% lower already at the upper end of normal postchallenge glycemia (6.5–7.7 mmol/L) 2 hours after the glucose challenge compared to the lower end of normal (51), suggesting not only a greater risk of developing diabetes in the future but also that low GI foods may be of benefit over larger glucose loads delivered by high GI foods, in these metabolically challenged individuals. The health benefits of low GI diets may extend to non-fatal chronic diseases as well as age-related macular degeneration and cataracts (52–54).

Regardless of the large body of evidence that low GI diets may have health benefits in chronic disease risk reduction, numerous controversies around the GI concept have restricted the use of the GI at the population level and in nutrition counseling. One issue is the variability observed between GI values for the same foods between different laboratories, which can be resolved by using the standard method of GI testing and calculations (27). GIs derived using the standard methodology should be preferentially chosen for dietary advice, for experimental purposes and epidemiological investigations. Small variability between GI values in GI testing is expected as in all dietary studies; however, despite this variability and the criticisms around the GI concept, low GI foods have been shown to successfully improve glycemic control in people with type 2 diabetes in many studies internationally (31, 34). These positive results would have been difficult to achieve should the GI concept be undermined significantly by ‘confounding factors.’ These results further suggest that low GI foods in mixed meals are effective. The GI concept has been considered too difficult to understand by the lay person. However, low GI dietary advice has been successfully used to lower the GI of the diet in individuals with type 2 diabetes (31). Key features of this advice include providing simple low GI food substitution lists and focusing on the lowest GI categories of foods, rather than focusing on conceptual explanations. The lowest GI categories of foods include beans, lentils, chickpeas, split peas, barley, pasta cooked al dente (i.e. slightly undercooked), and parboiled rice. Furthermore, low GI diets were found to be easier to follow and more palatable over diets based on carbohydrate counting in children with type 1 diabetes and their parents (55). Finally, low GI labels on carbohydrate food products may also aid consumers in utilizing the GI concept and hence in making informed decisions about the carbohydrate quality of foods (55).

### Carbohydrates and satiety

#### Dr. Alison L. Eldridge, Nestlé Research Center

In light of the growing obesity epidemic, many health conscious consumers are looking for foods and snacks that help keep hunger away and control overconsumption. One approach to help consumers better control their food intake may be to increase satiation, the factors that bring a meal to an end. Another approach is to enhance satiety, the feelings of fullness, and decreased hunger after a meal that suppresses the urge to eat.

There are several potential physiological mechanisms involved in satiation and satiety, each of which provides potential targets for products with these benefits (56). The act of chewing targets sensory aspects of satiety (57), the form of the food (e.g. texture) can alter satiety expectations (58), slowing the rate of gastric emptying may impact satiety (59), and foods or food components such as fiber that attenuate blood glucose may impact appetite and food intake (60). When measuring satiety in a research setting, the goal is to understand the influences on food intake to propose and evaluate solutions for product innovation and renovation. Tools to evaluate various aspects of satiety include sensory testing, subjective appetite evaluation, gastric imaging, and measurement of circulating satiety peptides, hormones, and nutrients. For weight control, the goals are to shorten the period of satiation and to increase the period of satiety. The type of question determines study execution, but typically food intake and subjective data are collected in a design that includes a preload followed by *ad libitum* intake of a meal.

Carbohydrates contribute a high proportion of calories in the diet, so understanding the effects that different carbohydrates have on satiety is of potential interest in helping consumers meet their needs. Overall, carbohydrates are considered to be intermediates between proteins and fats in promoting satiety, with proteins considered the most satiating of the macronutrients. Although the amount of total carbohydrate does not seem to relate to satiety (61), the type and physical form of carbohydrate does. For example, refined sugars are rapidly digested and appear to be less satiating than complex carbohydrates or fibers (62). Carbohydrates in beverage form appear to be less satiating than carbohydrates in food (63). While fiber increases satiety, different types of fibers influence satiety in different ways, with some fiber types increasing satiety and others having no effect (64). There are many proposed mechanisms for this effect, including chewing time, lower energy density, increased intestinal transit time, and fermentation. The European Food Safety Authority (EFSA) recently published guidance on scientific requirements for health claims related to appetite ratings, weight management, and blood glucose concentrations (65).

The ultimate objective for understanding the effects of different carbohydrates on satiation, satiety, and food intake is to help consumers make informed choices about what and how they eat. The EFSA has to date evaluated and rejected satiety claims for six types of fiber (66). Although a growing number of products are positioned for satiety in the marketplace, or are under development, they vary widely in the underlying scientific substantiation. Satiety claims should be based on the behavioral response as opposed to biochemical measurements, because if consumers are unable to perceive a difference in hunger or fullness, they will not be able to change their eating behavior. Understanding how consumers interpret satiety claims is critical to this discussion. It is also important to consider the potential benefits of different types of satiety claims and whether they are related simply to controlling hunger and appetite or if they are also associated with controlling food intake or even weight loss. Additional research is needed to characterize fibers as related to satiety, the role of food form and structure, combinations of various carbohydrate sources, and combinations of fibers with protein.

### Carbohydrate, mood, and cognition

#### Dr. David Benton, Swansea University

The brain is the most metabolically active organ in the body. Although it uses glucose as its major source of energy, it requires a continuous supply because its reserves are limited. The brains of children use glucose at a greater rate than that of adults. The adult brain accounts for 2% of body weight but 20% of the basal metabolic rate. As the carbohydrate in a meal delivers glucose into the blood stream, there has been an interest in the impact of this macronutrient on the brain, specifically on mood and cognition.

Although there is a popular belief that a sugar-containing drink in the short term results in a ‘rush,’ a feeling of energy, there is no experimental support for this idea. In the first hour after consumption of a sugar-containing drink, there are reports of small increases in subjective energy. However, the effects are small and not easily demonstrated (67). After several hours, meals high in carbohydrate result in reports of feeling less energetic (68). There are several reports that a diet generally higher in carbohydrate is associated with better mood (69). More specifically, in many individuals, a poor mood stimulates the eating of palatable high-carbohydrate/high-fat foods that have been found to enhance mood (69). De Castro et al. found that it is longer-term carbohydrate intake (over several days) that relates to mood (70).

The impact of sugar on the behavior of children became of interest in the consumer and scientific sectors in the early 1990s. In 1995, Wolraich et al. published a review of the impact of sucrose on behavior in children and found that irrespective of the test used or the behavior observed, there was no evidence of an adverse effect (71). The uniformity of these findings resulted in a decrease in additional scientific work on the topic. Later, an intervention trial found no evidence that sucrose influenced behavior after consumption by ‘sugar-reactive’ children (72). Another study showed that a 25-g sucrose drink provided in the afternoon resulted in more time spent on a task 15 min later (73).Consumption of palatable sugar-containing foods actually impacts release of endorphins – nature's morphine. In some populations studied, increasing intake of these foods decreases pain and induces a sense of calmness and well-being (74, 75).

There is increasing interest in the interaction between individual differences in the ability to control blood glucose levels (glucose tolerance) and the carbohydrate content of the meals. There is consistent evidence that those with poorer glucose tolerance have poorer memories (68). In children, memory and attention were better, and more time was spent on school work 2–3 h after a low-glycemic load breakfast (76). In adults, memory was better 2–3 h after a low-glycemic load breakfast. The addition of fiber to the diet or carbohydrate-containing food is one method whereby the GI may be reduced (77, 78).

Overall, the current data indicate that reactions to a meal depend upon the individual differences in glucose tolerance. The advantages for both mood and cognition can be seen with low amounts of carbohydrate or carbohydrates with a lower GI. Although there is some evidence that attention can be influenced, memory appears to be more susceptible to the carbohydrate content of meals.

### Update on prebiotics: new uses, new combinations, and new molecules

#### Dr. Pedro Antonio Prieto, Abbott Nutrition

Probiotics and prebiotics are two functional food concepts claiming to benefit or support gastrointestinal health through different mechanisms. Both concepts complement each other since a prebiotic is, in essence, a fermentation substrate used by probiotics. Formally a prebiotic has been defined as a non-viable food component, which confers a health benefit on the host associated with modulation of the microbiota (79). In turn, probiotics are the live microorganisms (microbiota) that provide beneficial effects to the host (80). It is worth emphasizing that the intestinal microbiota is a complex and dynamic system in which beneficial and harmful microorganisms coexist in a delicate balance that may be disrupted by disease, antibiotic use, and other environmental pressures. Indeed, it is recognized that among the functions of probiotics are inhibition of or competition with harmful bacteria, stimulation or modulation of immunity and enhancement of micronutrient absorption (81). The second concept, a more recent one, encompasses the notion of ingredients that are resistant to digestion, reach the colon, and are then selectively fermented, thus conferring benefits upon host health (82–84). Among other benefits, increased mineral absorption is a well-studied aspect of the combined effect of prebiotics and probiotics (17). Different categories of prebiotic ingredients are classified according to their origin and or molecular composition; some have already been well studied, such as certain oligosaccharides, and others are emerging. However, scientific explanations on their mechanisms of action and their clinical importance have been lagging behind their introduction to the marketplace.

A wealth of relevant research has emerged since the beginning of the 21st century and the resulting data point to modes of action and to the overall roles of natural and artificial oligosaccharides. It is now accepted that carbohydrates and their metabolism by bacteria are intimately related with the physiology and health of the human colon. In this account, biochemical modes of action of prebiotics are examined with emphasis on reports that emerged in the last 10 years. For example, recent discoveries on the dual role of bacterial surface proteins that function as adhesins and transporters for carbohydrate molecules, explain why human milk oligosaccharides have the ability to elicit *in vitro* growth and metabolism in characteristic human probiotics (85). In addition, combinations of prebiotics are now being used because, according to their size and composition, certain prebiotics may be hydrolyzed and fermented in the proximal colon and some others throughout the colon until the distal part. Several groups have now engaged in clinical research to assess their potential nutritional benefits in infants and children. Human milk oligosaccharides continue to be considered the gold standards of indigestible carbohydrate in pediatric nutrition as they comprise a heterogeneous and rich mixture of prebiotics (86), and studies on their ability to elicit growth and metabolic activity of bifidobacteria are also contributing to the understanding of plant oligosaccharides and synthetic prebiotics. Finally, a mixture of probiotics and prebiotics that improves the survival and implantation of live microbial dietary supplements in the gastrointestinal tract has been developed and studied, and this combination of ingredients is called a symbiotic (87).

Taken together, these novel contributions suggest that the next few years will produce even more convincing evidence on the physiologic roles and biological activity of fermentable oligosaccharides. Perhaps the study of diverse molecules and their metabolic destiny in the human colon will also shed light on the fundamental interactions between beneficial bacteria and their hosts.

### Fructose and health: is there evidence for harm?

#### Dr. John L. Sievenpiper, McMaster University and St. Michael's Hospital

Since Bray and Popkin (88) first linked fructose to the epidemic of obesity nearly 10 years ago, it has become a focus of intense concern regarding adverse effects on cardiometabolic risk factors. The scientific reports, more so commentaries and editorials, on the topic have led major diabetes (89) and heart (90) associations to caution against high intakes of fructose in their most recent recommendations. These recommendations, however, have been made in the absence of consistent clinical evidence of harm. The University of Toronto has conducted a series of Canadian Institutes of Health Research (CIHR)-supported systematic reviews and meta-analyses using Cochrane Collaboration methodology of controlled feeding trials examining the effect of fructose on cardiometabolic risk (91). Here, evidence for the adverse effects is reviewed briefly and then compared with the results of these systematic reviews and meta-analyses of human trials.

Fructose has been linked to adverse effects on blood lipids, uric acid, blood pressure, body weight, and glucose and insulin regulation. The evidence supporting these adverse effects is drawn mainly from highly reproducible animal studies. In two well-referenced examples, rodents show increased blood levels of triglycerides, insulin, free fatty acids, and increased insulin resistance and body weight (31, 92). In the absence of consistent evidence of harm in prospective cohort studies, the next line of evidence has come from poorly controlled ecological studies showing that the increases in fructose intake have paralleled the increases in overweight and obesity since the 1970s in the United States (88, 93, 94). Finally, human intervention studies with hypercaloric designs, in which fructose-containing beverages provide excess energy (+25% energy) at extreme doses relative to control diets, have been nominated as the highest level of evidence of harm. Whereas trials with isocaloric designs, in which fructose is exchanged with other sources of carbohydrate under energy-matched conditions, have failed to show consistent adverse metabolic effects, the hypercaloric trials have shown marked and consistent elevations in triglycerides and apolipoprotein B (95–97) and reductions in energy expenditure (98).

An increasing effect on de novo lipogenesis (DNL) is thought to be the principal mechanism underpinning the adverse effects of fructose. Classical biochemistry teaching is that fructose bypasses the control step of phosphofructokinase acting as an unregulated substrate for DNL (99). While it is well accepted that this mechanism operates in animal models, it is critical to note important differences in carbohydrate metabolism between animals and humans. In the mouse (up to 96%) and rat (>50%) (100–102), the percentage of carbohydrate intake that is shifted toward DNL is much higher than that for humans (approximately 5%) (103). There is also evidence in humans that catalytic amounts of fructose (≤10 g) improve glucose regulation by increasing glycogen synthesis and decreasing hepatic glucose production (104, 105). Methodology is another important consideration. Fructose is typically provided to animals at 60% of energy, which is a marked contrast to the median and 95th percentile of intake in humans of 10% and 20%, respectively (106). These factors make comparison of animal data to the human situation challenging. Whether the metabolic syndrome phenotype seen in animals can be induced in humans under conditions of ‘real-world’ fructose exposure is unclear.

Despite the uncertainties in the existing data, nutrition guidelines have taken a harm reduction approach to fructose. The American Heart Association recently put forth guidelines recommending restriction of added fructose for mitigation of chronic disease (107). The American Diabetes Association has also recommended against the use of added fructose as a sweetening agent owing to possible adverse effects on serum lipids (108). In both cases, the evidence cited to support these recommendations was drawn from only a selection of the >40 published feeding trials on this topic.

Contrary to the concerns expressed, the CIHR series of systematic reviews and meta-analyses have shown that there is a moderate body of consistent evidence drawn from more than 40 controlled feeding trials that fructose in isocaloric exchange for other sources of carbohydrate at low-to-moderate doses (<60 g/day, <10% of total energy) does not harm serum lipids (109), blood pressure (110), uric acid (111), body weight (112), or insulin (113) and may even benefit glycemia (113). Translation of these findings, however, is limited by the small sample sizes, short duration, and poor study quality of the available trials, as well as unexplained inter-study heterogeneity (lack of consistency in results owing to differences in study methodology) in some of the analyses.

Harm under certain conditions, however, remains an important consideration. An emerging body of consistent evidence shows that fructose consumed at high doses, such as >100 g/day across all groups (114) or >60 g/day in people with type 2 diabetes (109), or under hypercaloric feeding conditions (>+20% excess energy) may promote dyslipidemia, weight gain, and raised uric acid levels (115), although a principal role of excess energy cannot be excluded in the hypercaloric trials with formal tests of interaction suggesting that the adverse effects may be more attributable to excess energy than fructose. These data, however, are again limited by the small sample sizes, short duration, and poor study quality of the majority of the available trials, as well as some unexplained inter-study heterogeneity.

There remains a need for larger, longer-term, and higher quality ‘real-world’ feeding trials to disentangle the role of fructose from that of energy in cardiometabolic disease.

### Activities on functional foods and biomarkers in ILSI Europe

#### Agnès Meheust and Marie E. Latulippe, ILSI Europe

The European Branch of the International Life Sciences Institute (ILSI Europe) operates on the premise that global health promotion and disease prevention can only be achieved through evidence-based nutrition, founded on accurate data. The Functional Foods Task Force of ILSI Europe has contributed to the approach on how to substantiate health claims on foods. Both the FUFOSE (Functional Food Science in Europe) (116) and PASSCLAIM (Process for the Assessment of Scientific Support for Claim on foods) (117) projects have substantially contributed to this global discussion and outcomes have supported the development of the European regulation on nutrition and health claims (EC 1924/2006) (118). Indeed, in the context of functional foods and evidence-based nutrition, the FUFOSE project proposed a rationale for intelligent and strategic use of markers following specific criteria, developed the concept of factors and indicators, and pointed out the interest of using intermediates or surrogate markers. PASSCLAIM, in its guidance, emphasized the usefulness of markers of intermediate effects and the fact that the effects evaluated by the markers should be statistically and biologically meaningful, and introduced the idea that dose-related changes should be observed.

Numerous other initiatives addressed the question of providing guidance for marker selection, such as the Biomarkers Definitions Working Group (119) and the report commissioned by the US Food and Drug Administration to the Institute of Medicine on the evaluation of biomarkers and surrogate endpoints (120), among others. However, a critical cornerstone for this process remains to be clarified in detail: What are the criteria to guide scientists in the selection of markers?

Nutrition research is facing some challenges such as how to show that specific foods may have an impact on maintaining or improving specific health functions, whereas currently, nutrition effects are assessed via clinical endpoints linked to disease state. How much do markers used in a study reflect the clinical endpoint? How meaningful are these markers, mostly coming from the medical field, in assessing maintenance of normal body function?

All of these aspects should be considered when studying nutrition and health. Many articles or guidelines have highlighted that markers should be validated. However, no clear and practical framework has been proposed thus far for validation in the nutrition research field.

To stimulate research, innovation, and competitiveness, it is now time, in the European food industry, to provide nutrition scientists with clear guidance from public agencies on the specific type of marker information needed to scientifically substantiate an impact on health.

In 2011, ILSI Europe initiated an activity with the purpose of identifying consensus criteria for the evaluation of markers to use in nutrition research and to develop guidelines for the adequate use of these criteria. A scientific panel has been identifying the evidence-based validation criteria for markers based on a literature review. In parallel, a series of expert groups from various task forces of ILSI Europe have identified the criteria for marker validation based upon the markers broadly used in their expertise field of nutrition research. Data collected by the different groups will be reviewed and discussed with relevant experts, stakeholders, and representatives of other global initiatives to draw conclusions.

Understanding how and why markers can be considered as valid is a common concern. By creating a list of agreed-upon criteria, guidelines will be offered to the nutrition research field to harmonize marker selection and provide recommendations for the development of future markers. A list of validated markers will improve the ability to compare data across studies and ensure the highest quality of science-based nutrition information.

### Intestinal health: predictive biomarker for development of functional food

#### Dr. Gabriela Perdigon, CERELA-CONICET, Universidad de Tucumán

The intestinal ecosystem is a very complex network of interactions between prokaryotic-eukaryotic or eukaryotic-eukaryotic cells (121). In this microenvironment coexist in a perfect equilibrium a large number of bacteria (estimated to be more than 10^13^/g feces) and the immune cells associated with the mucosa responsible for the mucosal immune response. Many factors impact the gut microbiota, including nutrition, bacterial interactions, and presence of disease, pharmaceuticals, bacterial metabolites, antibodies, pH, age, and bile acids (122). We are just beginning to understand how probiotics impact immune activation. The balance between the microbiome and gut immune system can have important implications for systemic health such as food allergies and even colon cancer.

These characteristics of the intestinal ecosystem avoid the colonization of pathogenic bacteria (barrier effects) and it is difficult to modify its behavior by foods. However, functional food can influence not only the microbiota, favoring the growth of beneficial microorganisms, but also the mucosal immune system associated to the gut. These functional foods by definition can exert a beneficial effect in addition to their nutritional properties. They usually contain live microorganisms with probiotic characteristics as defined by the FAO (79). We have shown that *Lactobacillus casei* interacts with and activates epithelial cells to induce an immunoglobulin response (123–125). *L. casei* can, in effect, activate the immune system.

One of the important questions is how to select a functional food that will improve the intestinal health. The best option would be to select a good probiotic strain; however, what biomarker should be selected to indicate an impact on intestinal health? Potential options include cytokines, epithelial interleukin-6, immune cell receptor expression, or increased macrophage activity. Taking into account the beneficial effect of some probiotic strains present in functional foods, we analyzed the effect of a probiotic strain by *in vivo*, *ex vivo*, and *in vitro* assays comparatively with a non-probiotic strain. We established, by *in vitro* assay, that species specificity is not a necessary condition for the selection for the probiotic strain to be used in a functional food. The most predictive biomarkers for a beneficial effect on the gut are chemokine expression and macrophage activation. IgA producing cells and cytokine release are not exclusive to probiotic bacteria and therefore would not be proper biomarkers even in an *in vivo* assay. Our study also showed that probiotic characteristics are not related to the *L. acidophilus* lipoteichoic acid (LTA) molecule. However, the possibility that LTA acts together with other molecules in the immunomodulatory capacity of probiotic bacteria is not excluded. The next phase of this work is to demonstrate efficacy of these markers in humans.

### The intestinal microbiome and dietary patterns

#### Christian Hoffmann, University of Pennsylvania and Universidade Federal de Goiás

The intestinal microbiota, until recently considered a mere bystander, has been elevated almost to the status of an active organ, playing a role in a wide range of host physiological processes. Examples of these host–microorganism relationships are observed in the fermentation of indigestible carbohydrates, the synthesis of certain vitamins, the degradation of dietary oxalates, the development of the immune system, the protection of the epithelial tissue, and some angiogenesis stimulations (126). It is composed of >100 trillion microorganisms such as bacteria, archaea, micro-eukaryotes, and viruses, which are, for the most, not yet cultured (127). The combined genome of this microbial community is at least 10-fold greater than the human genome. The microbiota composition differs according to body site, and these differences are present not only between distinct body sites (such as mouth vs. skin) but also between regions that could be considered more similar to each other (such as regions of the gastrointestinal tract) (128). A distinct pattern can also be observed in the same body site according to health and disease state such as inflammatory bowel disease (129), obesity (130), and colon cancer (131). Several factors affect the composition and maintenance of the gut microbiome, including genetics and the host phenotype, the immune system (132), the intake of antibiotics, the environment, and the diet (133). Of these factors, diet, the main source of energy for the microbiome, is the easiest to manipulate in a clinical setting, with far fewer potential side effects.

Although the microbiota is different from person-to-person, individuals within a human population can be grouped according to their gut microbiome (134, 135). Indeed, thanks to dietary inventories coupled with next-generation DNA sequencing technology, a recent study showed that the human population could be distinguished in at least two groups, and within each group the microbiota is dominated by one or a few species. The species signature present in each cluster has been strongly associated with long-term dietary patterns. Particularly, the consumption of protein and animal fat was specific to the Bacteroides cluster, whereas carbohydrates consumption was associated with the *Prevotella*-dominated cluster. This confirms that diet can modulate the gut microbiota composition and therefore have an impact on human health; however, the mechanisms by which diet influences the gut microbiome remain to be fully understood.

Short-term dietary interventions have shown to be capable of causing a measurable effect on the gut microbiome composition within 24 h, but are not enough to completely change the system from a pre-existing state into another. Whether long-term interventions can do so is still an open question, but dietary therapies have the potential to be used to manipulate the host's associated microbiome. Applications are evident on the treatment of certain inflammatory bowel diseases, such as Crohn's disease. This new knowledge could also open the door to personalized therapies, in which treatments would be designed according to the subjects’ intestinal microbiome composition.

### Interaction between the intestinal microbiome and functional foods

#### Professor Robert A. Rastall, University of Reading

The idea that the vastly complex microbial ecosystem in the human colon has a profound impact on health is now gaining widespread acceptance in the scientific community and the food and healthcare industries. Recent research is illuminating the intricate interactions between microbes in the colon, their metabolites, and human metabolism, which will ultimately help to define ‘normal’ or ‘healthy’ gut microbiota.

Consequent to this area of work, there is increasing interest in the development of functional food ingredients to modulate the colonic microbiota and its metabolic profile for the promotion of health. Traditionally, this has been achieved through the administration of live bacterial supplements, or probiotics. Although there is accumulating evidence that specific, carefully selected bacterial strains can have health benefits, it is not possible to bring about large changes in the colonic microbiota in this way. In 1995, the concept of prebiotics was introduced. Since that time, there have been several definitions of prebiotics, with the most recent one published in 2010 (82). Essentially, prebiotics are food ingredients, supplements, or components that escape digestion in the small intestine and reach the colon largely intact. They are then selectively fermented by health-positive members of the colonic microbiota. All recognized prebiotics are carbohydrates with fructo-oligosaccharides (FOS), inulin, and galacto-oligosaccharides (GOS) being the only prebiotics in the European market. There are a much wider range of oligosaccharides recognized as prebiotics in Japan and many more are being investigated for their potential.

Although there is a range of prebiotics on the market, we have a poor understanding of the structure-function relationships in these molecules. Recent studies at Reading have given new insights into the influence of structure on prebiotic activity and can inform the development of new ingredients (136–138). Depending upon the prebiotic provided, the microbial populations, SCFAs produced and the products of microbial metabolism can be manipulated (139). Studies designed to examine differences in gut microbial response to fibers in lean vs. obese humans have shown an effect on acetate to propionate ratios with provision of oligodexdrins, but not with soluble glucose fiber or polydextrose (Kolida et al., 2012, unpublished data).

Pectins and oligosaccharides derived from them have been studied for their selectivity of fermentation and the effect on microbial populations depends upon the type of pectin provided (138).

Much of the early work in this area has focused on increasing the relative populations of bifidobacteria and lactobacilli. These are non-pathogenic species with a range of recognized health-positive attributes, including inhibition of intrinsic and extrinsic pathogens, modulation of immunity, and synthesis of vitamins. The health attributes of these organisms are still being explored in the laboratory and in human volunteer trials, and data are accumulating to support the use of prebiotics in a range of chronic and acute gut disorders. Recent thinking, however, has focused increasingly on the metabolites produced by the colonic microbiota and the impact of these metabolites on health is currently being studied. This widening of emphasis is stimulating debate over the definition of prebiotics.

There may be several mechanisms in operation producing the measurable differences in bacterial counts, and the extent to which each is in effect is not known. One such mechanism may be the inhibition of pathogen adherence to the host epithelial cell surface. In many cases, the first key step in the pathogenesis of disease-causing microbes is adhesion to a receptor target on the host cell. If such interactions can be blocked using receptor analogues (‘decoy oligosaccharides’) in foods, then resistance to food-borne pathogens may be increased. *In vitro* studies suggest that higher the degree of polymerization (DP) of GOS provided, the greater the inhibition of enteropathogenic *Escherichia coli* adhesion (140). Pectic oligosaccharides have similar effects on a number of pathogens (141). This area of research is still in its early stages and further progress is dependent on the economical and practical supply of candidate anti-adhesives. In addition, there is evidence that these carbohydrates inhibit pathogenic bacterial toxin production (140, 142).

The evolving view of prebiotic function, coupled with new insights into the structure-function relationships in prebiotic oligosaccharides, is providing opportunities or development of novel functional carbohydrates. Enzyme-based technologies are being brought to bear on the challenge of manufacturing enhanced forms of prebiotic food ingredients. Rational choice of synthetic catalyst and carbohydrates source can potentially allow the development of precisely targeted prebiotics with a higher degree of selectivity than the current generation. An increase in the number of bifidobacteria is considered a positive health effect by the EFSA. At the same time, there is still no known cause and effect as related to health outcomes in humans and much work is needed to understand the potential short- and long-term health benefits.

### Role of gut microbiota in obesity

#### Dr. Joel Faintuch, University of São Paulo

A microbiome refers to a collection of microorganisms associated with an organ or tissue. Colonization of the intestine begins at birth and continues throughout childhood. To give some perspective on the size of the gut microbiome, humans are composed of approximately 10 trillion cells; the microbiome is composed of 100 trillion germs. Once established, the microbiome may be acutely altered by illnesses, medications, or other temporary changes. With chronic modifications, the effect may be permanent. Diet is known to have early and important effects on the character of the gut microbiome (143).

The science around the gut microbiome and obesity has been under development for only a decade; therefore, much is yet to be uncovered about this relationship and the underlying mechanism(s) remain under investigation. Thus far, it has been documented that intestinal bacterial microbiota operate differently in the lean compared to the obese (144). For example, while four primary families exist in the human microbiome (Bacteroides, Firmicutes, Proteobacteria, and Actinobacteria), Firmicutes and Bacteroides seem to be most relevant in obesity. Similarly, a marked change in gut microbiota has been observed following surgical intervention for obesity (11).

Several observations to date suggest that successful manipulations of the gut microbiota may assist with weight loss. While artificially modifying the gut microbiota (such as with pharmaceutical suppression) may result in a weight change, the microbiota remains resistant to change and seems to revert to the original profile. In animal models, after 3 weeks of such suppression, modifications of the intestinal microbiota cause weight loss (145–147).

The study of microbes has added a new dimension to the vast and complex pathophysiology of obesity and diabetes. It seems that the microbial composition may impact glucose homeostasis and diabetes (148). Initially, the differences between the lean and obese were thought to relate to SCFA production. SCFAs are a source of leptin that inhibit appetite (149). An elevation of 20% of Firmicutes (which are higher in the obese) may equate to retention of 150 kcal/day in individuals given the same diet (143, 150). Now it is clear that there may be more complex factors, involving fermentation, modulation of intestinal absorption and the intestinal barrier, secretion of hormones related to appetite and insulin function (PYY, GLP-1, GLP-2), modulation of immunity and systemic inflammation, interference with topical deposition (obesity), and ectopic adipose tissue (steatosis) gene expression (151, 152).

Prebiotics and probiotics show promise for impacting some of the mechanisms under exploration. For example, by impacting the levels of PYY, GLP-1 and proglucagon to overall inhibit appetite and optimize glucose homeostasis (150). Additional work is needed, particularly human data, to confirm that these dietary components can have a clinically relevant impact on weight management.

### The relationship between intestinal microbiome and the central nervous system

#### Dr. Přemysl Berčík, McMaster University

The intestinal microbiota is a diverse and dynamic ecosystem that affects the host's metabolism and shapes intestinal motility and permeability, producing effects beyond the gut. An association of microbiota with behavior and the central nervous system (CNS) may initially seem far-fetched. Clinicians, however, have long recognized the benefit of antibiotics in the treatment of hepatic encephalopathy. Behavioral problems such as anxiety and depression are common comorbidities in patients with gut disorders. For example, 54%–94% of patients with irritable bowel syndrome (IBS) meet the criteria for at least one primary psychiatric disorder (153). Similarly, anxiety, and depression are estimated to affect 30% of patients with IBD during remission periods and 60%–80% during colitis flare (154–156). Higher cytokine levels (which are released during the inflammatory process or administered therapeutically) are associated with psychiatric symptoms and behavior changes (157–159). It should be also noted that chronic gastrointestinal disorders, such as IB or IBS, are associated with abnormal composition of gut microbiota (160).

Animal studies have shown that acute intestinal infections in mice are associated with anxiety-like behavior and activation of vagal pathways prior to onset of an immune response (161, 162). Chronic infection with *Helicobacter pylori* leads to abnormal feeding behavior and upregulation of central pro-inflammatory cytokines, which persist even after successful bacterial eradication (163). Low-grade gut inflammation induced by the non-invasive parasite *Trichuris muris* leads to anxiety-like behavior in mice, which can be normalized by treatment with an anti-TNFα (an inflammatory cytokine) agent etanercept or specific probiotic bacteria (164).

Recent studies have shown marked differences in behavior and brain biochemistry in conventional and germ-free mice, suggesting a crucial role of microbiota in the CNS function (165, 166). It has been shown that compared to germ-free mice, the mice colonized with commensal bacteria display anxiety-like behavior with differential expression of neurotrophins, such as BDNF and NGF, as well as multiple genes involved in the secondary messenger pathways and synaptic long-term potentiation in several areas of the brain (165, 166). Perturbation of gut microbiota by antimicrobials in healthy conventional mice increased their exploratory behavior and central neurotrophin levels (167); oral antimicrobials do not affect germ-free mouse behavior (167). It has also been demonstrated that colonization of germ-free mice with specific microbiota determines their behavior phenotype, modulating their exploratory behavior and brain chemistry (167).

Altogether, there is mounting evidence that the intestinal microbiota has the ability to affect the CNS and modulate its function via immune, neural, and likely metabolic mechanisms. Understanding of the microbiota–gut–brain axis will help us to better manage not only chronic gastrointestinal, but likely also psychiatric disorders. Specific probiotic bacteria and/or customized diets with prebiotics may then have a role in future therapies for these relationship disorders.

### Considerations related to the use of glycemic index and dietary fiber definition

#### Drs. Elizabete Wenzel de Menezes and Franco M. Lajolo, University of São Paulo and Food and Nutrition Research Center

Multiple activities were developed in 2011 by the Functional Foods Task Force of ILSI Brazil, in collaboration with scientists, health professionals, government, and industry, as well as nutrition students. These activities, which were based on a review of evidence, allowed for a scientific debate on implementation of the current Codex dietary fiber definition and on the feasibility of using the GI, and also demonstrated a need to discuss carbohydrates under a wider spectrum. The main recommendations of these activities are as follows:

1. In relation to implementation of the Codex dietary fiber definition and aiming at global harmonization (6, 7, 168, 169): a) consider inclusion of carbohydrates of DP 3–9, which are not hydrolyzed by the endogenous enzymes in the small intestine of humans, as dietary fibers; and b) achieve agreement as to which physiological effects can be scientifically linked to consumption of dietary fibers. For example, decreased blood total and/or LDL cholesterol levels, decreased postprandial blood glucose level, and increased stool bulk and/or decreased transit time, among others.
2. In relation to glycemic response (GI and GL), the following points were suggested (23, 26, 27, 43, 52, 54): a) recent research supports the idea that high postprandial glycemia can be linked to the progression of certain non-transmissible chronic diseases (NTCDs), including diabetes and CVD; b) diets rich in dietary fiber are important to decrease the risk of NTCDs and these benefits are enhanced when the intake of dietary fiber is associated with low GI foods; c) a significant portion of the scientific and health professional communities recognize the GI/GL concept and suggest its use; d) although methodological limitations exist, meta-analytical data indicate that low GI and GL foods may decrease disease risk and help control diabetes; and e) consumer education is important to promote the understanding and correct use of the GI/GL concept.

The presentations and discussions of the ‘Carbohydrates, Microbiome and Health’ event brought to the participants’ attention aspects of carbohydrates that are still rarely discussed in Brazil and also confirmed a few recommendations that were described above for dietary fiber and glycemic response.

## Conclusion

As one of three macronutrients, carbohydrates constitute a notable portion of energy intake; therefore, their impact on health is significant. This is made more complex by the variety of dietary carbohydrates available. In this workshop, the latest findings on various aspects of carbohydrates and health were reviewed. Key points are as follows:

1. It is clear that while there has been significant movement within Codex, efforts towards global harmonization of a science-based fiber definition are needed. This definition should account for the wide variety of physiological effects thus far demonstrated.
2. While varying by geographical region, demographics and forms of fiber consumed, fiber intake is generally quite low and added fibers may be useful in moving populations towards recommended intake levels.
3. Low GI foods can be used to modulate the postprandial glycemic response and may affect health outcomes. However, a number of variables can affect GI and there remains need for a good consumer use/communication tool.
4. Carbohydrate type may influence satiety and satiation. There is a need for additional work on measuring satiety, increasing consumer understanding of the concept, and assessing the impact of whole foods compared to ingredients.
5. Glycemic load and glycemic index, which can be influenced by the fiber content of a food, show links to memory, mood, and concentration in certain study populations although additional work is needed to show long-term effects.
6. The prebiotic effect of carbohydrates has long been known, but new research is clarifying the mechanisms underlying potential benefits. Key to demonstrating these benefits is the identification of validated biomarkers.
7. Beyond benefits of carbohydrates, sugars, particularly fructose, have drawn some negative attention. In this workshop, it was made clear that when human data are systematically compiled and reviewed, the effects observed in animal studies are not evident.
8. New research indicates that diet strongly influences the microbiome, and this may have implications for both disease and optimized health.
9. There is mounting evidence that the intestinal microbiota has the ability to impact the gut–brain axis. Understanding these relationships holds potential for managing both GI and psychiatric disorders.

Overall, there is much promise for development of functional foods that impact the microbiome and other factors relevant to health including glycemic response (glycemic index/glycemic load), satiety, mood, cognition, and weight management.
